# Scapular Dynamic Muscular Stiffness Assessed through Myotonometry: A Narrative Review

**DOI:** 10.3390/s22072565

**Published:** 2022-03-27

**Authors:** Ana S. C. Melo, Eduardo B. Cruz, João Paulo Vilas-Boas, Andreia S. P. Sousa

**Affiliations:** 1 Center for Rehabilitation Research—Human Movement System (Re)habilitation Area, Department of Physiotherapy, School of Health, Polytechnic of Porto, Rua Dr. António Bernardino de Almeida, 400, 4200-072 Porto, Portugal; ame@ess.ipp.pt; 2 Research Centre in Physical Activity, Health and Leisure, Faculty of Sport, University of Porto, Rua Dr. Plácido Costa, 91, 4200-450 Porto, Portugal; 3 Porto Biomechanics Laboratory (LABIOMEP-UP), University of Porto, Rua Dr. Plácido Costa, 91, 4200-450 Porto, Portugal; jpvb@fade.up.pt; 4 Center for Interdisciplinary Applied Research in Health, School of Health, Setubal Polytechnic Institute, Campus do IPS Estefanilha, 2914-503 Setubal, Portugal; 5 Department of Physiotherapy, School of Health, Setubal Polytechnic Institute, Campus do IPS Estefanilha, 2914-503 Setubal, Portugal; eduardo.cruz@ess.ips.pt; 6 Comprehensive Health Research Center (CHRC), Universidade Nova de Lisboa, 1169-056 Lisboa, Portugal; 7 Centre of Research, Education, Innovation and Intervention in Sport (CIFI2D), Faculty of Sport, University of Porto, Rua Dr. Plácido Costa, 91, 4200-450 Porto, Portugal

**Keywords:** myotonometry, scapular muscles, muscle mechanics, stiffness

## Abstract

Several tools have been used to assess muscular stiffness. Myotonometry stands out as an accessible, handheld, and easy to use tool. The purpose of this review was to summarize the psychometric properties and methodological considerations of myotonometry and its applicability in assessing scapular muscles. Myotonometry seems to be a reliable method to assess several muscles stiffness, as trapezius. This method has been demonstrated fair to moderate correlation with passive stiffness measured by shear wave elastography for several muscles, as well as with level of muscle contraction, pinch and muscle strength, Action Research Arm Test score and muscle or subcutaneous thickness. Myotonometry can detect scapular muscles stiffness differences between pre- and post-intervention in painful conditions and, sometimes, between symptomatic and asymptomatic subjects.

## 1. Introduction

Muscular stiffness, described as passive or dynamic [1,2,3], is a mechanical property that traduce the resistance offered to an action that leads to muscle tissue deformation [1,3]. More specifically, this muscular property derived from muscle structure and intrinsic material properties [4], namely from tendon [5], myofibrillar cross-bridges [5] (particularly titin filaments [6,7]) and muscular connective tissue [6]. The passive stiffness, commonly assessed with elastography methods [2,3], mainly represents the tissue adaptation [3] in it basal/passive status [8] and the baseline level of the stiffness [5]. The dynamic stiffness, assessed through myotonometry [9], is based on the free oscillation theory and results from the natural oscillation of the tissues, in response to a brief mechanical tap on the skin [10].

Both passive and dynamic stiffness are essential for adequate muscle contraction [7] and performance [11], as well as for adequate joint motor control [12] and integrity [13]. Muscle stiffness has been demonstrated to vary between subjects [14] according to age [12,15,16], muscle constitution, length, cross-sectional area [4,15] and measured point (myotendinous junction or muscle belly) [14]. Moreover, muscular stiffness has been demonstrated to be altered in conditions involving pain [17], injury [11], fatigue and cramps [18]. In pain conditions, the relevance of muscle stiffness in both movement and joint stability [19,20,21,22] highlight the possible influence of muscle stiffness deregulation, particularly in joints with high mobility like shoulder. Shoulder pain stands out for being a prevalent and recurrent of musculoskeletal condition [23,24] that involves stiffness adaptations in scapular muscles as upper trapezius (UT) [19,25,26]. These could be expected given the role of scapular muscles stiffness in shoulder stability [27] and function [28,29,30].

Muscle mechanical behavior has been studied for a long time [2,18]. In particular, muscular stiffness has been assessed by different non-invasive and reliable methods [1,2,3,4,18,31]:

(1) elastography [magnetic resonance (MR) [2], ultrasound shear wave [2,3] or strain [3]];
(2) tensiomyography [3];
(3) myotonometry [3].

Among the different methods, the ultrasound elastography [shear wave or strain [3,18], which only perform a qualitative assessment based in a color scale [2,3,32]] and the MR elastography [2,31,33] have the advantage of combining the assessment of passive stiffness [1,2,3] with operator visualization of the structures of interest [18,33]. However, these methods are associated with high costs and requires specialized operator’s knowledge [3,18] and more assessment time [34]. In turn, tensiomyography assesses muscle stiffness by considering maximal radial displacement [3,35] in response to a stimulated contraction [3,36] and requires several tools as electrical stimulator, data acquisition subunit, probe, electrodes, tripod with manipulating hand, and laptop for software interface [35,37]. The disadvantages of the previously mentioned methods has led to an increased interest in less expensive [1,3,4], easier to use [1,3,4] and less dependent technical expertise tools [1,3,4] to assess muscle stiffness in different conditions of muscles contraction. Myotonometry has been developed to fulfil these needs [3] by assessing the dynamic stiffness [1,18] of superficial soft tissues [4,9,18,33].

Considering that different methods measure different stiffness related variables [3], the growing use of myotonometry as a consequence of its advantages and the need of easily and regularly assess muscle mechanical properties in the rehabilitation settings, a review of this assessment tool is needed. This is particularly relevant for scapular muscles, once their impairment [38,39,40,41,42] as already been related to the long-term recovery and recurrence of shoulder pain [41,42]. Moreover, the lack of effectiveness reported by some studies [29,43,44,45,46,47], regarding scapular therapeutic approaches for shoulder pain, particularly therapeutic exercises, could be related with the necessity of considering other outcomes in the patient assessment process. Thus, the present study aims to review the psychometric properties and methodological considerations of myotonometry to assess muscular stiffness, particularly of the scapular muscles. To fulfil this purpose, this review is organized in four sections. In the first section the methodological requirements and limitations of myotonometry is presented. This section is followed by a section presenting the myotonometry psychometric properties, including validity, reliability, and responsiveness. The third section review the myotonometry applicability for assessing scapular muscles stiffness by synthesizing the previous studies. Finally, the conclusion section highlights the advantages of myotonometry for the assessment of muscular stiffness but warns of the cautions that should be considered.

## 2. Guidelines to Myotonometry Measurements of Muscular Stiffness and Obtained Data

**Figure 1:**
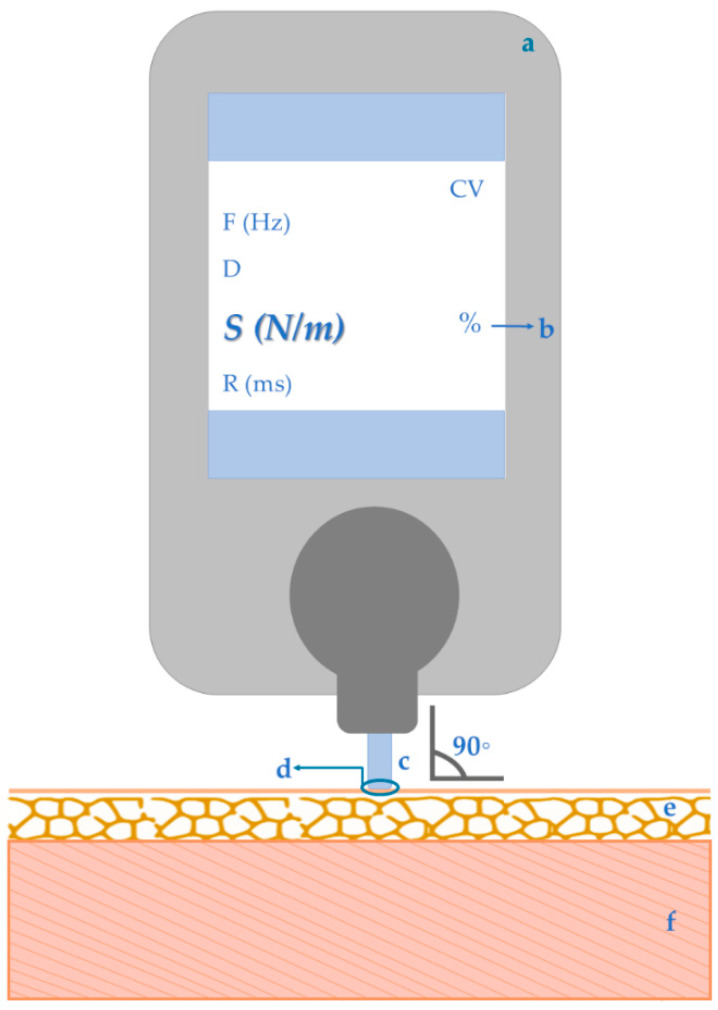
Myotonometry tool and its specifications: a—Equipment, b—Coefficient of variation, c—Probe function, d—Measurement point, e—Adjacent tissue, f—Eligible muscles.

Several requirements and limitations should be considered when using myotonometry, particularly MyotonPRO digital palpation device (MyotonPro, Myoton AS, Tallinn, Estonia), to assess muscle stiffness (Figure 1):

- Equipment:
  - Programming the data acquisition:
    - Introducing participant data (as weight, height, gender, and dominant side) [9]
    - Planning a “pattern composer”, this is, defining an assessment protocol regarding the muscles to include and their condition of assessment (rest or contraction), the subject position and the measurements side, location and nº of repetitions [9]
    - Uploading the participant and assessment data to the myotonometry tool
  - The assessor should guarantee the equipment’s stability and avoid the contact with external factors (as clothes) to not influence the device’s impulses neither the tissues oscillations [9];
- Coefficient of variation (total measurements’ variability according to subject, assessor and device accuracy): should be lower than 3% [9];
- Probe function: superficial tissues pre-compression followed by release of mechanical impulse and, consequently, muscular oscillation recording [1,3,4,9,18,43];
- Measurement point: superficial reference of the muscles of interest, based not only in previous studies using myotonometry [48], but also researches using tools as algometer [25,48] and electromyography [4,48,49]. For repeated measurements, the same measuring points as well as same muscular and environmental conditions (as time of the day and subject’s position), must be kept [9];
- Adjacent tissue: Measurement is only possible if the overlying subcutaneous fat is not higher than 20 mm [3,9,50];
- Eligible muscles: Superficial muscles [1,3,9], if bigger than 3 mm thickness and 20 g mass [9].

**Figure 2:**
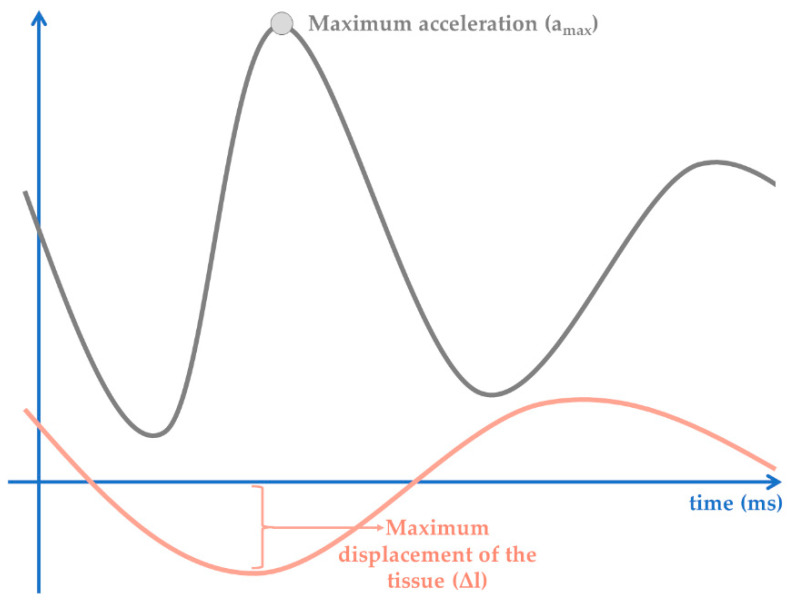
Graphical representation of the variables used in dynamic stiffness calculation.

Myotonometry assesses muscle mechanical properties, particularly, muscle dynamic stiffness traduced as [3,9,18]:

Dynamic stiffness (N/m) = a_max_·m_probe_/Δl

where a_max_ represent the maximum amplitude of the acceleration of oscillation (mG); m_probe_ represent probe mass and Δl represent the maximum displacement of the tissue (mm) [9] (Figure 2).

## 3. Myotonometry Psychometric Properties Regarding the Measure of Muscular Stiffness

### 3.1. Validity

There is no gold standard of stiffness measurement in the literature, making comparisons to an accepted standard difficult [51,52]. In the absence of a criterion for measuring stiffness [51,52], the validity of the myotonometry tool has often been determined by construct validity [51]. From these perspectives, myotonometry construct validity was done by comparison it with several non-stiffness variables as: (a) level of muscle contraction, for rectus femoris of healthy subjects (r^2^ = 0.9547) [52]; (b) static and dynamic strength measures, for soleus and lateral and medial gastrocnemius stiffness of healthy subjects [r = −0.81 to 0.48 (*p* < 0.05)] [53]; (c) lateral and palmar pinch strength and Action Research Arm Test score, for extensor digitorum and flexors carpi radialis and ulnaris of stroke patients [r = 0.25 to 0.52 (*p* < 0.05)] [54]; (d) muscle strength and muscle or subcutaneous thickness, for lower limb muscles of stroke patients [r = −0.84 to 0.46 (*p* < 0.05)] [55].

In turn, from the previously mentioned methods described to assess muscle stiffness, to our knowledge, only ultrasound shear wave elastography has been used to assess myotonometry validity.

The validation of myotonometry, particularly of Myoton as an instrument to measure muscular stiffness, by the correlation with this method when comparing muscular stiffness variables [1,4,18,49,56], has been done for several muscles [1,4,18,49,53,56], with different locations and functions [42,57,58,59,60,61]. In this case, the correlation values ranged from −0.25 [1] to 0.71 [4] for healthy participants [1,4,18,49,56] (Table 1). Only one study reported no correlation between the two measures [1]. This study assessed relative changes in upper trapezius muscle stiffness between pre and post eccentric exercise.

Despite the concurrent validity of myotonometry against elastography is the more frequently adopted approach, the differences between these two methods [1,3] should be considered in the analysis of the results presented in Table 1. Although both shear wave elastography and myotonometry use the principle of Young’s modulus, the measured variable may depend on the method used [4]. The differences between the two methods are summarized in Table 2. There are variations such as the type of stiffness measured [dynamic or passive stiffness [1,3,62]], the depth of measurements [1] [superficial muscular stiffness measured with myotonometry, may not be comparable to the smaller and deeper measurements provided by shear wave [4]], but also in the related reliability of the variables measured [3,4,7,25,48,56,63,64,65].

### 3.2. Reliability

Myotonometry reliability was already assessed for several muscles of different body segments [3,4,7,21,25,48,56,63]. Most studies only included healthy subjects [3,4,21,25,48,56,63], only one study included participants with musculoskeletal disorders in their sample [7].

The reliability values range from 0.229 [25], for UT, to 1 [4], for erector spinae. Specifically, regarding the scapular muscles and, in this case, the trapezius muscle, high to very high reliability were found for its three portions [7,21,25,48,63], with the exception of one study that reported a low to high reliability for the upper trapezius [25]. A more detailed description of reliability values for different muscles is presented in Table 3.

As can be seen in Table 3, muscle stiffness assessment with myotonometry has been already studied in two muscular conditions, at rest and during contraction. The inclusion of these two conditions in muscle stiffness assessment protocols could be important given the dynamic characteristic of the soft tissues [74] and the influence of muscular length in the afferent inputs coming from muscle receptors [16]. Moreover, it could be useful to verify whether, particularly in subjects with conditions as pain, the relation of the muscle stiffness with the number of activated crossbridges is maintained [75].

### 3.3. Responsiveness

Responsiveness is a psychometric property traduce as the ability of an instrument to detect a meaningful change, in a clinical state, over time [54,76]. Regarding myotonometry, only one study was inferred about this property [54], demonstrating that the extensor digitorum, the flexor carpi radialis, and the flexor carpi ulnaris dynamic stiffness in stroke patients improved after intervention (robot-assisted training, mirror therapy, mirror therapy with mesh-glove electrical stimulation, or conventional rehabilitation). In the mentioned study [54], great sensitivity for change was found for the affected limb (−0.71 to −0.83) but not responsiveness for the unaffected limb (−0.42 to −0.48).

## 4. Applicability of Myotonometry for Assessing Scapular Muscles Stiffness

Several muscles have been assessed with myotonometry [1,4,7,14,18,25,49,56], however to our knowledge, among the scapular muscles, only the trapezius muscle has been assessed [1,7,14,21,25,26,63,66,68,77,78].

The trapezius is a standout muscle for scapular stabilization that act in a strong relation, mainly, with the major scapular mover—the serratus anterior [79]. In shoulder pain conditions, both have been reported as possibly altered, namely by decreased and/or timing changed activation of lower trapezius (LT), middle trapezius (MT) and serratus anterior (SA) [38,41,79,80] or by increased [38,39,40,41,42,79] or decreased UT activity [81,82,83]. Impairments in the activity of levator scapulae [19,84,85] and pectoralis minor [84,85,86] muscles were also reported.

**Figure 3:**
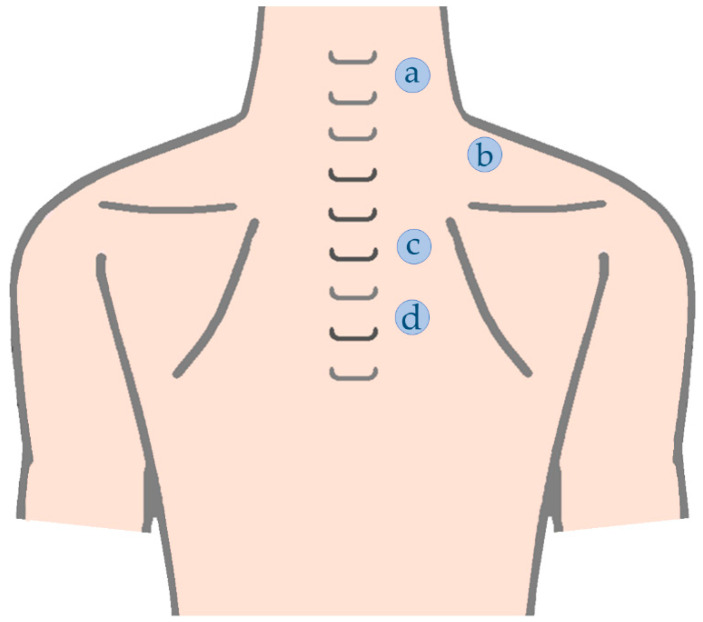
Trapezius muscles assessment points: (a) upper trapezius C5/6 level; (b) upper trapezius C7 level; (c) middle trapezius; (d) lower trapezius.

**Figure 4:**
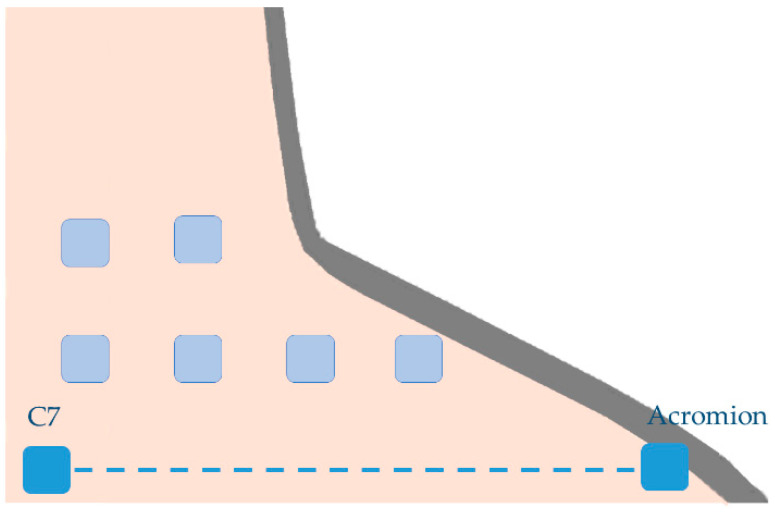
Upper trapezius muscles grid of measurement points.

Previous studies regarding myotonometry, had already presented muscular assessment point references for the trapezius portions [7,12,21,25,87,88,89] (Table 4 and Figure 3). However, a study about a 3D model construct through magnetic resonance [90] recommended other superficial references for upper trapezius, which might also be interest to consider given the possibility of considering some fibers with a more vertical orientation [79,90,91] compared with the “traditional” reference that possibly represent fibers with horizontal orientation [91,92] and higher cross-sectional area [91] (Table 4 and Figure 3). In addition, some studies [1,14,63] that assessed UT stiffness, measured this outcome through a grid of measurement points covering an extended area of UT muscle. Thus, considering the distance between C7 spinous process and the acromion several measurement points, separated by 1/6 [1,14,63] and/or 1/7 [14,63] of the mentioned distance, were defined. These measurement points include both muscle belly and myotendinous sites once muscle stiffness could be dependent on the location of the measurement point [1,63] (Figure 4).

**Figure 5:**
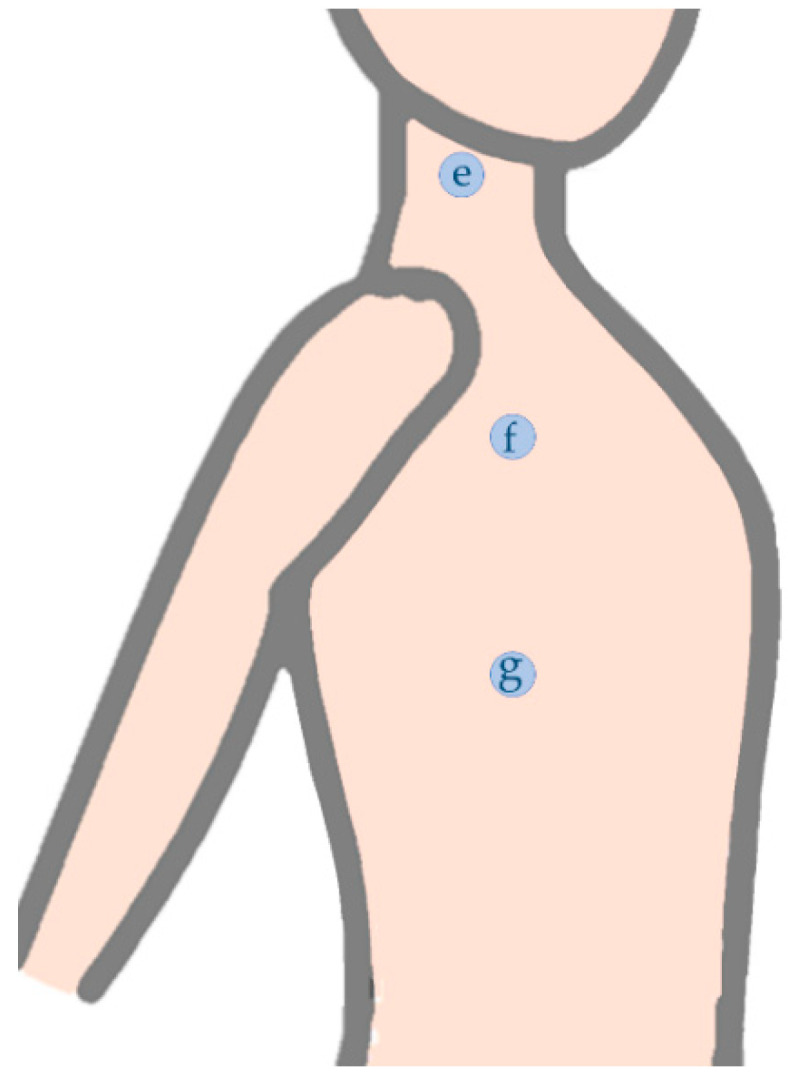
Serratus anterior and levator assessment points: (e) levator scapulae; (f) SA upper/middle portion; (g) SA lower portion.

The superficial references for serratus anterior assessment with surface electromyography [93,94,95,96] could be considered for myotonometry assessment, once studies with ultrasound or magnetic resonance imaging [97,98,99] report thickness values similar to the trapezius muscle [97,98,100,101,102], from 4.3 mm at rest to 11.8 mm while contracting [99]. Moreover, the studies about muscular thickness reported that lower trapezius is the thinner scapular muscle, ranging from 3.9 mm at rest [102] to 9.3 mm while contracting [98]. The fact that the myotonometry probe is placed on the assessment point, for each assessment repetition, with the patient already in the assessment position, also avoids the bias related to the proximity of the latissimus dorsi or pectoralis major [93] or to the geometric displacement (given skin movement during upper limb motions) [103] that could happen during surface electromyography [93]. As in the case of upper trapezius, serratus anterior could benefit form being assessed in two different portions, the upper/middle [93,94,96] given its role in the scapular protraction [93] and the lower [93], given its higher participation in scapular upward rotation [93,103] (Table 5 and Figure 5).

In its turn, the assessment of muscular stiffness of levator scapulae and pectoralis minor, that was already done with shear wave elastography [22,64,72,108,109], may not have been done with myotonometry given that these muscles are deeper positioned [19,110]. However, considering that a reference to the assessment of levator scapulae has been used to collect surface electromyography [90,104,105] and that it thickness ranges from 4.15 mm at rest to 6.38 mm while contracting [101,111], this muscle seem to fulfill the requirements for myotonometry assessment (Table 5 and Figure 5). However, future studies are required to confirm this possibility.

### Myotonometry Ability for Measuring Differences or Changes in Muscular Stiffness in Pain Conditions Involving Scapular Muscles

The relevance of scapular muscle stiffness to the shoulder complex and the possible muscle’s stiffness changes resulting from the scapular position and their influence in muscular length [64] had led to the development of studies comparing trapezius stiffness, measured through myotometry, for between group comparisons as well pre and post intervention comparison [14,26,66,68,77,78].

UT stiffness was compared between subjects, or body sides, with and without pain conditions (Table 6). While two studies [26,77] reported significant differences between groups, by comparing subjects with different upper trapezius pain levels (0 to 3 in VAS) [26] or by comparing symptomatic and asymptomatic moderate neck pain subjects [77], 3 other studies found no differences in UT stiffness both between pain and healthy subjects [14,66,68] and between the affected and the non-affected extremity of the same subject [66,68].

The trapezius stiffness comparison of pre- and post-intervention moments has already been made for several rehabilitation techniques (Table 6). Four studies [14,25,68,78] report significant differences between the assessment moments traduced into a reduction of UT stiffness after treatment. However, the opposite results were found by Sokk et al. [66] for UT stiffness and by Kisilewicz et al. [25] considering MT and LT stiffness.

## 5. Points That Need to Be Addressed in Future Studies

**Figure 6:**
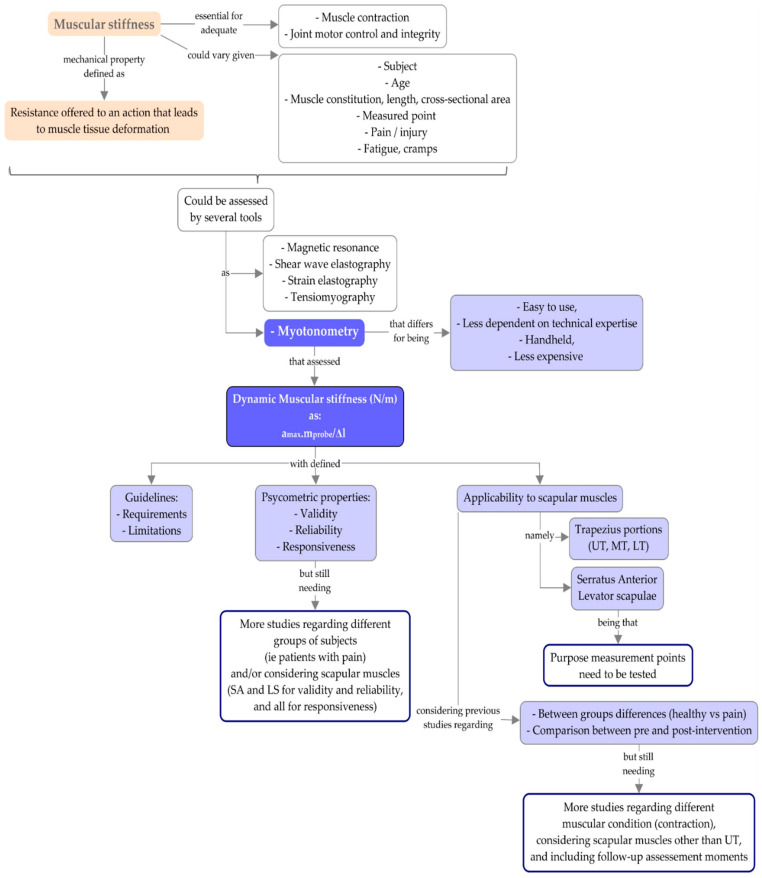
Summary of narrative review information regarding scapular dynamic muscular stiffness assessment through myotonometry and identification of the issues to be considered in future studies (LS: levator scapulae; LT: lower trapezius; MT: middle trapezius; SA: serratus anterior; UT: upper trapezius).

The summary of the information gathered in this review is presented in Figure 6. However, despite the several studies mentioned in the present review and their different aims, future studies regarding myotonometry, particularly for scapular muscles are still needed. Specifically, studies assessing the following issues are required:

- Myotonometry assessment of serratus anterior and levator scapulae muscles are needed to validate the purposed assessment points, to define the myotonometry psychometric properties considering these muscles and to increase the knowledge about these muscles’ mechanical properties.
- The myotonometry psychometric properties should also be researched in subjects with different conditions, such as pain.
- The use of myotonometry not only at rest condition but also during contraction, could bring new information that could help to standout adaptations in muscle stiffness modulation, given the muscular activity required and variation in the range of motion used in this muscular condition [112,113,114].
- In studies with the intention to infer about intervention effects, the inclusion of follow-up moments could help to understand whether stiffness changes will be kept over time.

The aspects that should be considered in future studies are summarized in Figure 6.

## 6. Study Limitations

The present narrative review has limitations. First, for being a narrative review, the present study could present some possible bias given the absence of predefined hypothesis and protocol-based (also considering data extraction and synthesis), the lack of necessity of following guidelines (as the ones purposed by PRISMA) or to the reduced database consulted during search. Although this review presents the guidelines for the correct use of myotonometry, it should be considered that these are specific for one equipment. Moreover, given the equipment limitation regarding the possible interference of subcutaneous fat, it would be important in future studies consider the measurement of subcutaneous fat in the specific measurement point of each muscle of interest as a criteria to use myotonometry.

## 7. Conclusions

The advantages of myotonometry together with the well-defined guidelines, the mostly high to very high values of reliability, the inferred responsiveness regarding the affected limb of stroke patients and its possible applicability to assess different scapular muscles stiffness, seems to support its use as a non-invasive method in the assessment of muscular mechanical properties as stiffness (N/m), for clinical practice or research. However, caution should be taken given the variable or no correlation with elastography, even if this may be justified by differences in the outcome measured.

## Figures and Tables

**Table 1:** Myotonometry validity data of correlation with shear wave elastography.

Muscle	Correlation Values		
	Values (*p* Value)		Classification
Upper trapezius	r = −0.25 to 0.50 (*p* > 0.05) [1]	Not statistically significant	
Infraspinatus	r = 0.35 to 0.37 (*p* < 0.05) [4]	Fair	
Rectus femoris	r = 0.398 to 0.416 (*p* < 0.05 and *p* < 0.01, respectively) [56]		
Biceps brachii	r = 0.479 to 0.583 (*p* < 0.05) [49]	Fair to Moderate	
Gastrocnemius	r = 0.463 to 0.71 (*p* < 0.05 or *p* < 0.01) [4,18,56]	Fair to Good	
Erector spinae	r = 0.51 to 0.54 (*p* < 0.05) [4]	Moderate	
Biceps femoris	r = 0.594 to 0.652 (*p* < 0.01) [56]		
Tibialis anterior	r = 0.540 to 0.561 (*p* < 0.01) [56]		

Legend: Correlation values classification—no correlation if values <0.25, fair if 0.25–0.5, moderate to good if 0.5–0.75, and good to excellent if >0.75 [4].

**Table 2:** Comparison between myotonometry and shear wave elastography for muscular stiffness assessment.

	Shear Wave Elastography	Myotonometry
Instrument characteristics	• Objective [4,18] • Non-invasive [1,4,18]	
	Real-time [1,3,64] Required technical expertise [18]	Less expensive [1,4] Handheld [1,3,4] Easy to use [1,3,4,66]
Structures assessed	Deep [1,4]	Superficial [1,4]
Type of stiffness measured	Passive [1,3]: resistance to elongation or shortening or, in physical terms, the change in tension per unit change in length [67]	Dynamic [1,25,68]: resistance to a force that deforms muscle initial shape [3,25,68]
Measurement mode	Elastic [4]/shear [3] modulus, that uses ultrasound radiation forces [4]	Damped oscillation method following a dynamic transformation of the muscle in response to a short-term external mechanical impulse [69]
Measurement process	Transducer parallel to the muscle fibers [1,4,18]—wave travel horizontal (along fibers [15]) to the point of application through tissue [4] Transducer held stationary for 10 s with minimal pressure applied on the skin [1,3,4]—acoustic radiation force to perturb muscle tissues [70] Measurement estimate based on the velocity of ultrasound propagation from an entire defined region of interest [3,4,18] and based on tissue density [15]—converted into Kpa values through Young’s modulus formula for every pixel [3,71]	Probe perpendicular to the skin surface [1,3] Constant pre-compression force (0.18 N) in the underlying tissues, followed by a short mechanical impulse (0.4/0.6 N for 15 ms) [1,4,18] Recording of muscle oscillation [4], reflecting viscoelastic properties of the tissue [3] Data by computational software, calculated from the acceleration of the testing probe during oscillations [3]
Measurement Interpretation	Velocity of shear waves (proportional to shear modulus [64]) rise with increase in passive muscle stiffness [1,64]	Higher values of dynamic stiffness imply more energy to modify the shape of the tissue [3]
Scapular muscles Assessed	In healthy subjects: UT [1,22,64,65,72] MT, LT and SA [64] Levator scapulae [22,64,72] In pain conditions: UT [1,22]; Levator scapulae [22]	In healthy subjects: UT [1,7,14,21,26,63] In pain conditions: UT [7,14,25,26,66] MT and LT [25]
Results SWE vs. Myotonometry	Myotonometry presented lower coefficient of variability [4] and similar values of reliability compared to SWE Myotonometry present high to very high reliability for upper limb, lower limb and spine muscles [3,4,25,56] in healthy subjects, and low to very high reliability for UT in both healthy [7,21,25,48,63] or with a musculoskeletal disorder subjects [7]; while SWE present moderate to very high reliability for upper limb, lower limb and spine muscles [3,4,56,64,65] in healthy subjects. Myotonometry present ability to discriminate between different muscle contraction intensities, but the same did not happen always for SWE [4]	

Legend: dynamic stiffness (DS); healthy subjects (HS); maximum amplitude of the acceleration of oscillation (amax); maximum displacement of the tissue (Δl); middle trapezius (MT); lower trapezius (LT); pain conditions (PC); probe mass (mprobe); shear wave elastography (SWE); upper trapezius (UT).

**Table 3:** Myotonometry reliability data.

Muscle	Sample	Assessment Conditions			Reliability Values	
		Assessment Moment	Rater	Muscle Condition	ICC Values	Classification
Upper trapezius	Healthy and MSKd	IS	Inter-rater	Rest	0.97 [7]	Very high
	Healthy	IS	Intra-rater	Rest	0.86 [48]	High
		BD			0.229 [25] to 0.86 [63]	Low to very high
		IS	Inter-rater	Rest and contraction considered together	0.97 [21]	Very high
		BD	Intra-rater		0.97 [21]	Very high
Middle trapezius		BD	Intra-rater	Rest	0.813 to 0.963 [25]	High to very high
Lower trapezius		BD	Intra-rater	Rest	0.820 to 0.926 [25]	High to very high
Infraspinatus		IS	Intra-rater	Rest	0.98 [4]	High to very high
				Contraction	0.98 [4]	High to very high
Erector spinae		IS	Intra-rater	Rest	1 [4]	High to very high
				Contraction	0.99 to 1 [4]	High to very high
Rectus femoris		IS	Intra-rater	Rest	0.938 [56]	Very high
				Contraction	0.872 [56]	High
Vastus Lateralis		IS	Intra-rater	Rest	0.97 [3]	Very high
		BD			0.93 [3]	Very high
Medial gastrocnemius		IS	Intra-rater	Rest	0.904 [56] to 1 [4]	Very high
				Contraction	0.856 [56] to 0.99 [4]	High to very high
Biceps femoris		IS	Intra-rater	Rest	0.884 [56]	High
				Contraction	0.861 [56]	High
Tibialis anterior		IS	Intra-rater	Rest	0.880 [56]	High
				Contraction	0.894 [56]	High

Legend: BD: between days; IS: intrasession; MSKd: musculoskeletal disorders; Reliability values classification—little, if any reliability, if values <0.15, low if 0.16–0.49, moderate if 0.50–0.69, high if 0.70–0.89, and very high if values >0.90 [73].

**Table 4:** Description of the trapezius muscle assessment points.

Muscle of Interest	Measurement Points
**Upper trapezius C5/6 level**	At the level of C5/C6 about 2 cm lateral from the midline [90]
**Upper trapezius C7 level**	Mid-way between C7 spinous process and the angle of acromion [7,12,21,87,88,89]
**Middle trapezius**	Mid-way from T4 spinous process to the medial border of spine of the scapulae [25]
**Lower Trapezius**	Mid-way from T6 spinous process to the medial border of spine of the scapulae [25] OR Mid-point of the lateral border of the fibers of lower trapezius [25]

**Table 5:** Description of the serratus anterior and levator scapulae assessment points.

Muscle of Interest	Measurement Points
**Levator scapulae**	Between the posterior margin of sternocleidomastoid and anterior margin of the upper trapezius [104,105,106,107], at level of C4/5 [90]
**SA upper/middle portion**	Over the fourth rib, at the midpoint between the latissimus dorsi and the pectoralis major [93,94]
**SA lower portion**	Over the seventh rib, in the midline of the axilla [93], for SA lower portion (SAlow) [93]

**Table 6:** Myotonometry ability to identify differences or changes in scapular muscles stiffness in pain conditions (✓ for *p* < 0.05; X for *p* > 0.05) and the respective groups, muscle assessed and values of muscle stiffness (mean and SD) and *p* value.

	Study Objective		Group	Muscle Assessed	$\bar{X}$	SD (N/m)	*p* Value
Mild (until 3 in VAS) UT pain (20.83 ± 1.12 years old) [26]	BGc	✓	VAS 0	UT (muscle belly)	170.56	28.45	*p* < 0.05 *, for VAS 3 in comparison with other 3 groups
			VAS 1		161.67	16.59	
			VAS 2		160.48	20.72	
			VAS 3		191.50	25.74	
	IE	-	-				
Moderate work-related neck disorders (30–55 years old) [77]	BGc	✓	Pain	UT (C5/6 and C7 level)	301.50	23.50	*p* = 0.006 *
			Control		270.90	33.70	
	IE	-	-				
Unilateral chronic shoulder pain together with, at least, 2 sensitive sites (myofascial trigger points) (18–70 years old) [68]	BGc	X	Control (Us, before)	UT (trigger points)	324.42	11.39	*p* = 0.057
			Control (Us, after)		334.68	11.10	
			Pain (before)		332.32	10.97	
			Pain (after)		300.66	9.43	
	IE	✓ for Myofascial trigger-point Release	Pain (before vs. after)				*p* = 0.012 *
Long-standing, nonspecific and nontraumatic neck-shoulder pain (20–61 years old) [14]	BGc	X	Control (MB sites)	UT (15 adjacent points)	237.80	42.8	*p* = 0.273, for comparison of both measurement sites
			Control (Mt sites)		327.50	55.9	
			Pain (MB sites before)		258.70	41.10	
			Pain (Mt sites before)		330.40	50.8	
	IE	✓ for Eccentric Training	Pain (MB sites after)		226.80	20.00	*p* < 0.001 *, for comparison in both measurement sites
			Pain (Mt sites after)		287.30	47.80	
Subacromial impingement syndrome (49.20 ± 9.48 to 50.90 ± 9.10 years old) [78]	BGc	-	-				
	IE	✓ for Thoracic mobilization and/or Extension exercise	Pain (TM before)	UT (center of muscle belly)	257.90	29.03	*p* = 0.001 *
			Pain (TM after)		232.50	20.49	
			Pain (exercise before)		257.70	19.33	*p* = 0.001 *
			Pain (exercise after)		236.10	27.27	
			Pain (TM plus exercise before)		257.50	25.61	*p* = 0.001 *
			Pain (TM plus exercise after)		223.00	32.83	
Stage II or III of unilateral frozen shoulder syndrome (38–74 years old) [66]	BGc	X	Control (Us, before)	UT (center of muscle belly)	≈235	_	*p* > 0.05
			Control (Us, 1 m after)		≈215	_	
			Control (Us, 6 m after)		≈200	_	
			Pain (before)		≈240	_	
			Pain (1 m after)		≈225	_	
			Pain (6 m after)		≈220	_	
	IE	X for Manual manipulation (under anaesthesia)	Pain (before vs. after)				*p* > 0.05, for comparison in each assessment moments
Unilateral neck or shoulder pain and active myofascial trigger points in the trapezius muscle (19.8 ± 2.4 years old) [25]	BGc	-	-				
	IE	✓ for Ischemic compression	Pain	UT (distally of muscle belly’s center)	232.00	29.70	*p* = 0.03 *
				UT2 (proximally of muscle belly’s center)	269.00	42.10	
				MT	405.30	192.10	*p* = 0.40
				LT (mid-point)	347.50	110.40	*p* = 0.29
				LT (lateral border mid-point of muscle fibers)	331.70	89.30	

Legend: ≈: when stiffness values were only presented in the original study through a graphic; 1 m: one month; 6 m: six months; After: stiffness values measured after intervention; Before: stiffness values measured before intervention; BGc: between groups comparison; IE: Intervention effects (comparison between pre and post-intervention moments); LT: lower trapezius; MB: muscle belly sites; Mt: myotendinous sites; MT: middle trapezius; SD: standard deviation; TM: thoracic mobilization; Us: unaffected side; UT: upper trapezius; VAS: visual analog scale; $\bar{X}$: mean.

## Data Availability

Not applicable.
